# Effect of androgens on Sertoli cell maturation in human testis from birth to puberty

**DOI:** 10.1186/s12610-021-00150-8

**Published:** 2021-12-14

**Authors:** Marion Lapoirie, Frederique Dijoud, Hervé Lejeune, Ingrid Plotton

**Affiliations:** 1 Université Claude Bernard Lyon 1, Lyon, France; 2 Service de Médecine de la Reproduction et Préservation de la Fertilité, Hôpital Femme Mère Enfant, Bron, France; 3 Institut de pathologie Multisite des Hospices Civils de Lyon, Site Est, Boulevard Pinel, Bron, France; 4 Inserm, U1208 Bron, France; 5 Service de Biochimie et Biologie Moléculaire, Université Claude Bernard Lyon1, INSERM 1208, Groupement Hospitalier Est, Centre de Biologie et Pathologie Est, 59, Boulevard Pinel, 69677 Bron, Cedex France

**Keywords:** Cellules de Sertoli, Barriere hémato testiculaire, Récepteur aux androgènes, Connexine-43, Spermatogenèse- Hormone Anti-Mullerienne, Androgènes, Sertoli cells, Blood, Testis barrier, Androgen receptor, Connexin-43, Spermatogenesis- anti-Mullerian hormone, Androgen

## Abstract

**Background:**

Androgens are well known to be necessary for spermatogenesis. The purpose of this study was to determine Sertoli cell responsiveness to androgens according to age from birth to puberty.

**Results:**

Testicular tissue samples were studied in a population of 84 control boys classified into seven groups according to age**:** group 1 (1–30 days), group 2 (1–3 months), group 3 (3–6 months), group 4 (0.5–3 years), group 5 (3–6 years), group 6 (6–12 years), and group 7 (12–16 years). We compared these data with those of 2 situations of pathology linked to androgens: 1/premature secretion of testosterone: 4 cases of Leydig cell tumor (LCT) in childhood; and 2 /defect of androgen receptors (AR): 4 cases of complete form of insensitivity to androgen syndrome (CAIS). In control boys, AR immunoreactivity (ir) in Sertoli cells appeared between 4.6 and 10.8 years of age, Anti-Mullerian Hormone (AMH) ir in Sertoli cells disappeared between 9.2 and 10.2 years of age. Connexin 43 (Cx43) ir in Sertoli cells and histological features of the onset of spermatogenesis appeared between 10.8 and 13,8 years of age. Cx43 ir was significantly higher in 12–16 year-olds than in younger boys. In case of CAIS, no spermatogenesis was observed, both AR and Cx43 ir were undetectable and AMH ir was elevated in Sertoli cells even at pubertal age. In the vicinity of LCTs, spermatogenesis occurred and both AR and Cx43 ir were strongly positive and AMH ir in Sertoli cells was low for age.

**Conclusions:**

Androgen action on Sertoli cells is required for onset of spermatogenesis and premature androgen secretion by LCT can induce spermatogenesis in the vicinity of the tumor. AR ir appeared earlier than onset of spermatogenesis, with large interindividual variability. The timing and mechanisms of Sertoli cell responsiveness to androgens are important issues for understanding the induction of spermatogenesis at puberty.

## Résumé

**Contexte:**

Les androgènes sont bien connus pour être nécessaires à la spermatogenèse. Le but de l’étude était de déterminer l’évolution de la réactivité des cellules de Sertoli aux androgènes en fonction de l’âge depuis la période néonatale jusqu’à la puberté.

**Résultats:**

Des échantillons de tissu testiculaire ont été étudiés dans une population de 84 garçons témoins classés en 7 groupes selon l’âge: groupe 1 (1–30 jours), groupe 2 (1–3 mois), groupe 3 (3–6 mois), groupe 4 (0,5–3 ans), groupe 5 (3–6 ans), groupe 6 (6–12 ans), groupe 7 (12–16 ans). Nous avons comparé ces données avec celles de deux situations de pathologies liées aux androgènes: 1/ une sécrétion prématurée de testostérone: 4 cas de tumeur à cellules de Leydig (LCT) dans l’enfance; 2/ une résistance aux androgènes par mutation du récepteur aux androgènes (AR): 4 cas de forme complète de syndrome insensibilité aux androgènes (CAIS). Chez les garçons témoins, l’immunoreactivité (ir) au AR dans les cellules de Sertoli est. apparue entre 4,6 et 10,8 ans, l’ir de l’hormone anti-mullerienne (AMH) dans les cellules de Sertoli a disparu entre 9,2 et 10,2 ans. L’ir de la connexine 43 (Cx 43) dans les cellules de Sertoli et les caractéristiques histologiques du début de la spermatogenèse sont apparues plus tard entre 10,8 et 13,8 ans. L’intensité de Cx 43 ir était significativement plus élevée chez les 12–16 ans que chez les garçons plus jeunes. Dans les cas de CAIS, aucune spermatogenèse n’a été observée, AR ir et Cx 43 ir étaient indétectables et AMH ir restait élevée dans les cellules de Sertoli à l’âge de la puberté. En outre à proximité des LCT, il est. observé une initiation de la spermatogenèse; AR ir et Cx43 ir étaient franchement augmentées et AMH ir dans les cellules de Sertoli était faible pour l’âge.

**Conclusions:**

L’action des androgènes au niveau des cellules de Sertoli est. nécessaire pour initier la spermatogenèse. De plus, une sécrétion prématurée d’androgènes, comme dans la situation de cas de LCT, est. capable induire une spermatogenèse à proximité de la tumeur. AR ir apparait un peu avant le démarrage de la spermatogenèse, il existe cependant avec une grande variabilité interindividuelle. L’apparition d’une réponse aux androgènes apparait comme un paramètre important à évaluer pour améliorer la compréhension de l’induction de la spermatogenèse.

## Introduction

Androgens are well known to be necessary for spermatogenesis. The period from birth to puberty is poorly studied to evaluate the establishment of spermatogenesis in humans. It would be interesting to study the role of androgens in the kinetics of the development of spermatogenesis.

In adults, spermatogenesis duration is 74 days [1], close to the duration of the only successful in vitro production of human spermatozoa from spermatogonia of adult seminiferous tubules [2]. However, induction of complete spermatogenesis at puberty can be estimated by the interval between the first signs of puberty (onset of testicular growth at 10.6–11 years [3]) and the presence of spermatozoa in the ejaculate at 13–15 years [4]. It seems to be far longer, probably because the first wave of spermatogenesis involves a process of testis maturation with the constitution of the blood/testis barrier and apoptosis of a large number of germ cells before completion of spermatogenesis.

In adults, Sertoli cell stimulation by testosterone, locally produced by Leydig cells under the control of LH, is required for spermatogenesis. At minipuberty, Sertoli cells do not express androgen receptor (AR) and are unable to promote germ cell maturation despite the presence of FSH, LH and testosterone [5]. Onset of AR expression in Sertoli cells seems to be a critical step for initiation of spermatogenesis [6].

The aim of this study was to determine and characterize the evolution of Sertoli cells responsiveness to androgens according to age from birth to puberty by studying specific markers: AR, anti-Mullerian hormone (AMH) and connexin 43 (Cx43).

Testicular tissue samples were collected in population of 84 control boys aged 0 to 16 years classified into 7 group according to age and compared between two situations of pathology linked to androgens: 1/ premature secretion of testosterone in childhood in case of Leydig cell tumor (LCT); and 2/ defect of androgen receptors (AR) in complete androgen insensitivity syndrome (CAIS). We reported tissue organization and cell content, onset of AR immunoreactivity (ir), decrease in AMH ir as a marker of the effect of testosterone on Sertoli cell [7], and Sertoli cell Cx43 ir as a marker of spermatogenesis. This enabled study of the role of androgens on Sertoli cell function and spermatogenesis, and describing the dynamics of testis maturation from birth to puberty. The conditions of spermatogenesis induction in prepubertal tissues are discussed.

## Materials and methods

### Patient selection

Patient records from June 1993 to December 2019 were identified by computerized search of our pathology register at the Lyon University Hospital. We excluded the following groups: neonates born at less than 37 weeks’ gestation or with intrauterine growth retardation, patients with testicular history (cryptorchidism, testicular atrophy or chemotherapy), patients with pathology of penis development (micropenis or hypospadias) and patients with other endocrine diseases. We excluded poor sample quality with autolysis. Tissue samples were collected at necropsy (mainly carried out for sudden infant death syndrome) or from biopsy carried out for preservation of fertility before sterilizing cancer treatment or surgical exploration of testicular mass; these biopsies were taken at a distance from testis tumor, none of which were Leydig cell tumors. Samples were divided into seven age groups, in order to target the major stages of testicular development. We defined the groups according to previous studies and the Nistal classification [8]: Group 1, newborns (1 to 30 day-old neonates), Group 2, mini-puberty (1 to 3 month-old infants), Group 3 end of mini-puberty (3 to 6 month-old infants), Group 4, early childhood (6 months to 3 years old), Group 5, 2nd childhood period (3 to 6 year-old boys), Group 6, onset of puberty (6 to 12 year-old boys), and Group 7 adolescence (12 to 16 year-old boys).

We also analyzed samples of testicular tissue located in the vicinity of a Leydig cell tumor (LCT) in 4 patients (three 6 year-olds and one 10 year-old) and samples of testicular tissue from 4 patients with complete androgen insensitivity syndrome (CAIS) aged 3 months, 14, 18 and 20 years. In three cases, point mutations (p.R779W, p.R586C and p.R616H) were observed, inducing amino acid change. Conversely in the fourth case, the mutation p.Q35X induced complete absence of the AR protein.

Following institutional rules, all necropsies and testicular biopsies were authorized by the Local Ethics Committee of our University Hospital. Written consent from the closest relatives was obtained in all cases. Autopsies were carried out within 24 h (Collection declaration: DC-2008-72 / DC-2020-3919).

### Antibodies and immunochemistry

Testicular tissues were studied in the Pathology Department at Lyon University Hospital. Tissue samples were fixed at least 24 h in 4% neutral buffered formalin (*n* = 67) or acetic formalin alcohol (AFA) (*n* = 25). After paraffin embedding, tumor specimens were cut in 3 μm- thick sections and stained routinely with HPS (Hematoxylin, Phloxine, Safran). Immunohistochemistry staining was performed employing the streptavidin-biotin and peroxidase method on an automated immunohistochemical system (Benchmark XT, Ventana Medical Systems Inc., Tucson, AZ, USA) according to the manufacturer’s instructions and using the reagents supplied with the kit. Briefly, after deparaffinization and rehydration, sections were subjected to antigen retrieval (Heat-Induced Epitope Retrieval [HIER] method, 8 min at 100 °C, pH 6). Tissue sections were covered with H_2_O_2_ to block endogenous peroxidase, followed by an additional washing procedure with the manufacturer’s buffer, and pretreated by ULTRA CC1 (ULTRA Cell Conditioning Solution, Ventana Medical Systems Inc.). Slides were then incubated with one of the following antibodies for Sertoli cell markers: AMH, a monoclonal mouse antibody (Clone 5/6 ACRIS AMO5878SU, dil. 1/50, 32 min); AR, a monoclonal rabbit antibody, (Clone EPR1535(2) GeneTex, dil. 1/50, 32 min); or Connexin-43 (Cx43), a polyclonal rabbit antibody (SIGMA HPA035097, dil. 1/500, 32 min). After washing, tissues were incubated with biotinylated antibodies, followed by the streptavidin-biotin complex, the amplification reagent, and the streptavidin-peroxidase conjugate. Tissue staining was visualized with a DAB substrate chromogen solution (Ultraview ref. 700–500 DAB detection, Ventana). Counterstaining was performed using hematoxylin and Bluing Reagent (Ventana Medical Systems Inc., 4 min). Positive controls were performed by using biopsies of adult men with obstructive azoospermia.

### Histological analysis [9]

Two pathologists (L.M, D.F) blind to the clinical data independently performed slide evaluation on a Leica DM2500 microscope. For analysis, five image fields at X10 magnification were taken from each section. All cords/tubules within these five image fields were evaluated and scored.

The number of seminiferous cords/tubules per sample was counted. The diameter of the seminiferous cords/tubules was measured on round sections. The cords/tubules were considered as being round if the ratio of the longer to the smaller diameter of the tubule was ≤1.5. The presence of a lumen and the most advanced germ cell were noted. Germ cells were identified on the basis of their morphology (size, shape) and location [10]. The germ cell content in the testis was evaluated by 2 methods, both measured from 100 cord/tubular sections (the maximum number of cord/tubule sections available for the 14 samples containing 50–99 cord/tubule sections): 1) number of spermatogonia per seminiferous cord/tubule transverse section, referred to as S/T score [9], and 2) percentage of the seminiferous cord/tubule cross-sections containing at least one germ cell, referred to as the Tubular Fertility Index (TFI) [11].

The number of Leydig cells was estimated on three fields at 40x magnification, and classified as rare (< 15) or numerous (≥15).

### Immunostaining analysis

The following scoring approach in the assessment of immunostaining was used: 0 = no or non-specific cell staining; score 1 = weak (intensity) and incomplete (quality) staining of > 10% of cells (quantity); 2 = moderate complete staining of > 10% of cells; 3 = strong complete staining of > 10% of cells [12].

### Statistical analysis

Median seminiferous cord/tubule diameter, S/T score, TFI and immunostaining scores were compared between groups by non-parametric Kruskal-Wallis test followed by Mann-Whitney test for pairwise comparison.

Presence of advancing germ cells, presence of lumen in seminiferous tubules and Leydig cell amounts (rare versus numerous) were compared between groups by Fisher’s exact test.

Since transition from prepubertal to pubertal state of markers could be modeled as a sigmoid curve [13], the point of inflexion of the sigmoid curve was calculated for AR ir, AMH ir, Cx43 ir, seminiferous cord/tubule diameter, S/T score, presence of a lumen in at least some seminiferous tubules, and presence of maturing germ cells (i.e., beyond spermatogonia).

## Results

### Samples

**Fig. 1:**
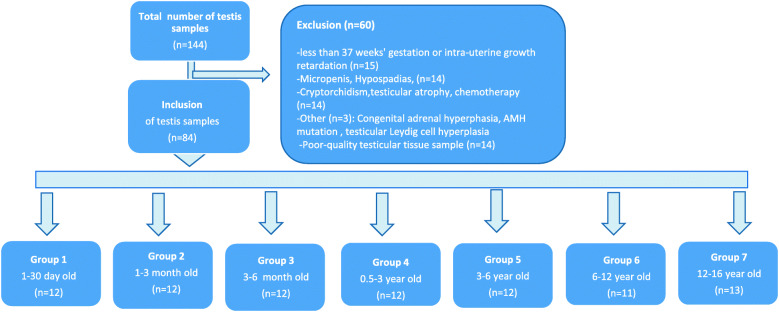
Flowchart of testis sample inclusion between 1993 to 2019. Determination of exclusion criteria and definition of 7 age groups studied in the protocol. *n* = number of samples in each group

From June 1993 to December 2019, 144 testicular samples were collected, from boys aged 1 day to 16 years. We excluded 60 samples (Fig. 1), including 14 for poor quality, and analyzed only slides without any seminiferous epithelium detachment from basal membranes indicating autolysis. Finally, 84 samples were selected for this study. Tissue samples were collected at necropsy (*n* = 43) or from biopsy carried out for preservation of fertility (*n* = 18) or surgical exploration (*n* = 23). We included samples in the different groups according to age: Group 1, newborns (*n* = 12), Group 2, mini-puberty (*n* = 12), Group 3 end of mini-puberty (*n* = 12), Group 4, early childhood (*n* = 12), Group 5, 2nd childhood period (*n* = 12), Group 6, onset of puberty (*n* = 11), and Group 7, adolescence (*n* = 13). Seventy-eight samples (groups 1–7, CAIS and LCT) contained more than 100 seminiferous cords/tubules, and 14 samples (groups 1–7) contained between 50 and 100. The context and number of seminiferous cords/tubules per group are given in Table 1.

**Table 1:** Characteristics of the testis samples for the 7 age control, Complete Androgen Insentivity Syndrom (CAIS) and Leydig Cell Tumor (LCT) groups

Groups	Groups 1–7	Group 1	Group 2	Group 3	Group 4	Group 5	Group 6	Group 7	CAIS	LCT
Age range	0–16 y.	0–30 d.	1–3 m.	3–6 m.	6 m. - 3 y.	3–6 y.	6–12 y.	12–16 y.	3 m.- 20y	6-10y.
n	84	12	12	12	12	12	11	13	4	4
Context										
Sudden death (n)	43	11	10	12	8	2	0	0	–	–
Peritumoral (n)	23	1	2	0	4	3	4	9	–	4
Fertility preservation (n)	18	0	0	0	0	7	7	4	–	–
No seminiferous tubules										
> 100 (n)	70	11	12	12	10	10	5	10	4	4
50–100 (n)	14	1	0	0	2	2	6	3	0	0

The Table gives the number of samples obtained in different contexts (sudden death, peri-tumoral or fertility preservation) and the number of samples with 50–100 or > 100 tubules, according to group (age groups 1–7, CAIS and LCT)

For CAIS, samples were obtained at therapeutic orchidectomy

*n* = number of samples (one sample for one subject); *y* year, *d* days, *m* month

### Germ cells and seminiferous cords/tubules

As presented in Table 2, cord/tubular diameter, S/T score, TFI, lumen and most advanced germ cell were significantly different in group 7 (12–16 y.o.) compared to the other age groups. CAIS samples exhibited a prepubertal pattern with mean tubular diameter lower than in 0–12 y.o. control boys and no lumen within the seminiferous cords. Spermatogonia were observed only in the youngest case (3 months old) and were lost in the other cases (14, 18 and 20 years). Despite their young age (6–10 years old), LCT samples exhibited a pubertal pattern with mean tubular diameter, S/T score and TFI as high as in 12–16 y.o. control boys, presence of a lumen in the seminiferous tubules, and post-meiotic germ cells. It was noticeable that the spermatogenic process was more advanced in the vicinity of the tumor.

**Table 2:** Results of histological data for the 7 ages control, Complete Androgen Insentivity Syndrom (CAIS) and Leydig Cell Tumor (LCT) groups

Groups	Groups 1–7	Group 1	Group 2	Group 3	Group 4	Group 5	Group 6	Group 7	CAIS	LCT
Age range	0–16 y.	0–30 d.	1–3 m.	3–6 m.	6 m. - 3 y.	3–6 y.	6–12 y.	12–16 y.	3 m.- 20y.	6-10y.
Mean tubular diameter (μm)										
n	84	12	12	12	12	12	11	13	4	4
median	75.0	75.0	75.0	80.0	75.0	65.0	65.0	170.0	57.5	137.5
[min-max]	[50–275]	[60–80]	[70–90]	[70–90]	[60–90]	[55–75]	[50–140]	[75–275]	[55–70]	[105–175]
Kruskal-Wallis/Mann-Whitney	*p* < 0.001	b	bc	c	bc	a	ab	d	a	d
S/T score										
n	79	12	12	12	9	12	10	12	4	4
median	1.96	2.77	1.42	1,41	1.46	1,27	2.16	12.07	**	2.60
[min-max]	[0.4–28.2]	[0.88–6.42]	[0.68–4.58]	[0.4–3.06]	[0.88–2.2]	[0.86–3.88]	[0.66–5.08]	[2.84–28.2]	[0–2.1]**	[2,2–3.34]
Kruskal-Wallis/Mann-Whitney	*p* < 0.001	ab	ab	ab	a	ab	ab	c	**	b
Testicular Fertility Index										
n	79	12	12	12	9	12	10	12	4	4
median	84.0	85.0	82.0	74.0	76.0	72.0	85.5	100.0	**	100.0
[min-max]	[34–100]	[50–99]	[40–100]	[34–90]	[58–94]	[42–100]	[42–100]	[88–100]	[0–95]**	[98–100]
Kruskal-Wallis/Mann-Whitney	*p* < 0.001	a	a	a	a	a	a	b	**	b
Most advanced germ cell										
Spermatogonia (n)	73	12	12	12	12	12	10	3	1**	0
Spermatocyte (n)	2	0	0	0	0	0	0	2	0	0
Round spermatid (n)	2	0	0	0	0	0	1	1	0	2
Elongated spermatid (n)	4	0	0	0	0	0	0	4	0	1
Spermatozoa (n)	3	0	0	0	0	0	0	3	0	1
Fisher’s exact test	*p* < 0.001									
Lumen										
Absent (n)	71	12	12	12	12	12	9	2	4	0
Present in only some tubes (n)	4	0	0	0	0	0	2	2	0	3
Present (n)	9	0	0	0	0	0	0	9	0	1
Fisher’s exact test	*p* < 0.001									
No Leydig cells										
rare (n)	35	2	0	1	10	12	9	1	0	4 *
numerous (n)	49	10	12	11	2	0	2	12	4	0 *
Fisher’s exact test	*p* < 0.001									

The Table gives the histological characteristics of the samples in each group for the following parameters:

- median tubular diameter (cord/tubular diameter)

-S/T score: number of spermatogonia per seminiferous cord/tubule transverse section,

- TFI: Tubular Fertility Index (percentage of the seminiferous cord/tubule cross-section containing at least onegerm cell),

- most advanced germ cell :it were significantly different in group 7 (12-16 y.o.) compared to the other age groups. CAIS samples exhibited a prepubertal pattern with mean tubular diameter lower than in 0-12 y.o. control boys and no lumen within the seminiferous cords.

Groups labeled with the same letters were not significatively different on pairwise comparison. P<0.05 wasconsidered as significant. (Kruskal-Wallis/Mann-Whitney)

n= number in the sample

min-max = minimum-maximum

### Leydig cells

**Fig. 2:**
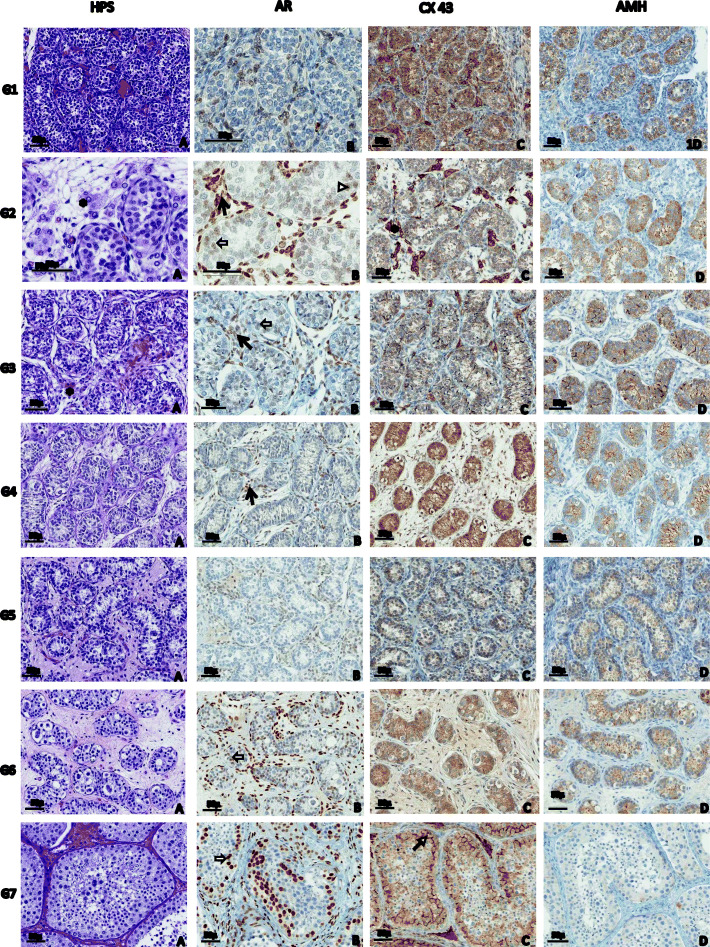
Histological and Immunostaining analysis of 7 age groups studied in the protocol (Gr1; Gr2; Gr3; Gr4; Gr5; Gr6; Gr7) for 4 conditions. HPS staining: Hematoxylin, Phloxine, Safran -Immunohistochemistry staining specific: Androgen Receptor (AR); Connexin 43 (CX43); Anti-Mullerian Hormone (AMH). Morphological aspects and immunohistochemistry profile with AR, Cx43 and AMH in the 7 age groups of normal testis. The seminiferous tubules have a prebubescent appearance (small tubules without lumen and spermatogonia) in group 1 to 5 (1A, 2A, 3A, 4A, 5A). In group 6, the tubules lengthen, central lumen become visible (6A). Leydig cells (*) are observed during minipuberty (2A, 3A) and more numerous at puberty (7A), immunostained by Cx43 (2C, 3C). No Leydig cell is observed in the other age groups (1A, 4A, 5A, 6A). Expression of AMH is specific by Sertoli cells decrease with age (1D, 2D, 3D, 4D, 5D 6D) and is almost zero at puberty (7D). Membranous staining of CX43 in Sertoli cells (←) is only present at puberty (7C). The AR expression in Sertoli cells (⇐) is very weak during minipuberty (2B), then turns negative (3B, 4B, 5B) to gradually reappear with significant expression in group 6 (6B) and strong expression in group 7 (7B). Peritubular cells (◊) are marked by immunostaining for AR in all age group (1B, 1B, 3B, 4B, 5B, 6B, 7B). *: Leydig cells,: peritubular cells nuclear AR staining membranous Cx43 staining

**Fig. 3:**
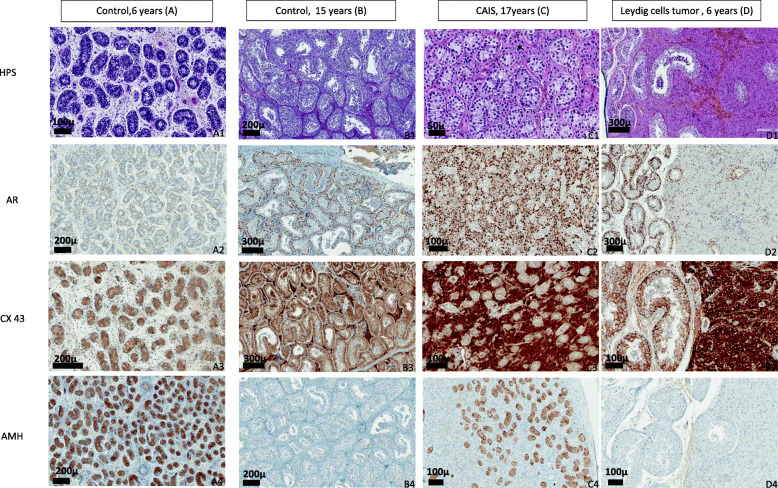
Histological and Immunostaining analysis of control groups compared to Complete Androgen Insensitivity Syndrome (CAIS) and Leydig Cell Tumor (LCT) groups for 4 conditions. HPS coloration: Hematoxylin, Phloxine, Safran. Immunohistochemistry staining specific: Androgen Receptor (AR); Connexin 43 (CX43); Anti-Mullerian Hormone (AMH)

Leydig cells were present but few during minipuberty (groups 2–3), decreased during childhood (groups 4–6) and increased at puberty (group 7) in the control boys (Fig. 2). In CAIS samples, Leydig cells were as numerous in the minipuberty (groups 1–3) as in the pubertal (group 7) groups (Fig. 3). In LCT samples (Fig. 3-D1), Leydig cells were rare or absent in the non-tumoral tissue, similarly to the childhood groups (groups 4–6) (Fig. 2).

### Sertoli cells

**Fig. 4:**
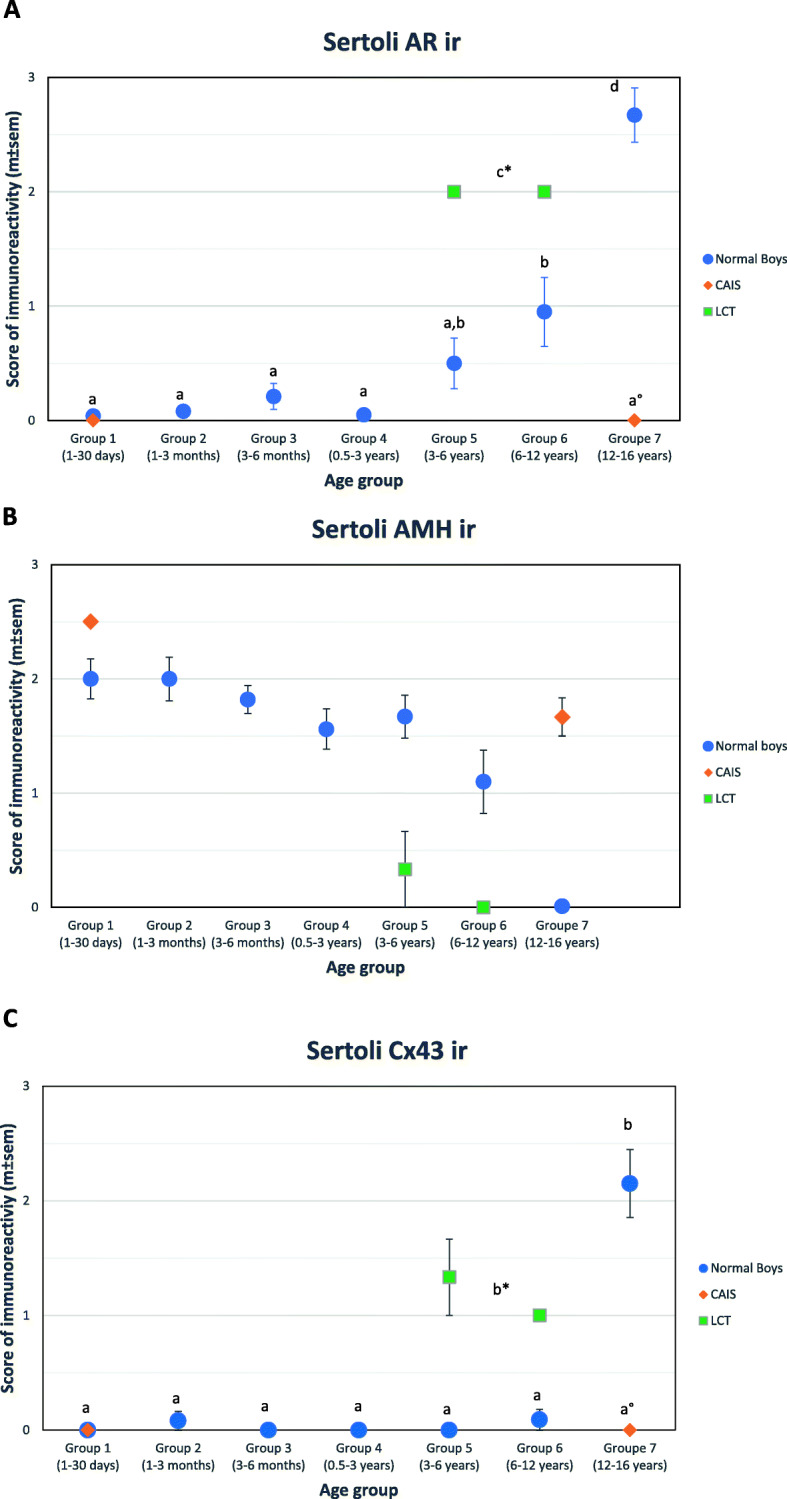
Immunostaining analysis of 7 ages of control groups studied in the protocol (Gr1; Gr2; Gr3; Gr4; Gr5; Gr6; Gr7) and compared to Complete Androgen Insensitivity Syndrome (CAIS) and Leydig Cell Tumor (LCT) groups. Semi-quantitative analysis of immunoreactivity (ir) of 3 specific Sertoli markers. AR (Androgen Receptor); CX43 (Connexin 43); AMH (Anti Mullerian Hormone). *P* < 0.05 was considered as significant. Figures 4-A, 4-B, 4-C: groups labeled with the same letters (a, b, c) are not significantly different on Mann-Whitney test for pairwise comparison

A very faint AR ir was observed in rare Sertoli cells in a small number of boys (7/38) during minipuberty (3–188 days). No AR ir was observed in Sertoli cells during childhood, until 4.6 years of age in our set. AR ir in Sertoli cells began to be positive in some boys (5/12) in group 5 (3–6 y.o.), increased significantly in group 6 (6–12 y.o.) and increased even more in group 7 (12–16 y.o.) (Fig. 4A, Fig. 2-7B).

AMH ir in Sertoli cells (Fig. 4B) was high during minipuberty and childhood (groups 1–5), decreased significantly in group 6 (6–12 y.o.) and disappeared in group 7 (12–16 y.o., Fig. 2-7D).

Membranous Cx43 ir in Sertoli cells (Fig. 4C) was significantly detected only in group 7 (12–16 y.o., Fig. 2-7C).

**Fig. 5:**
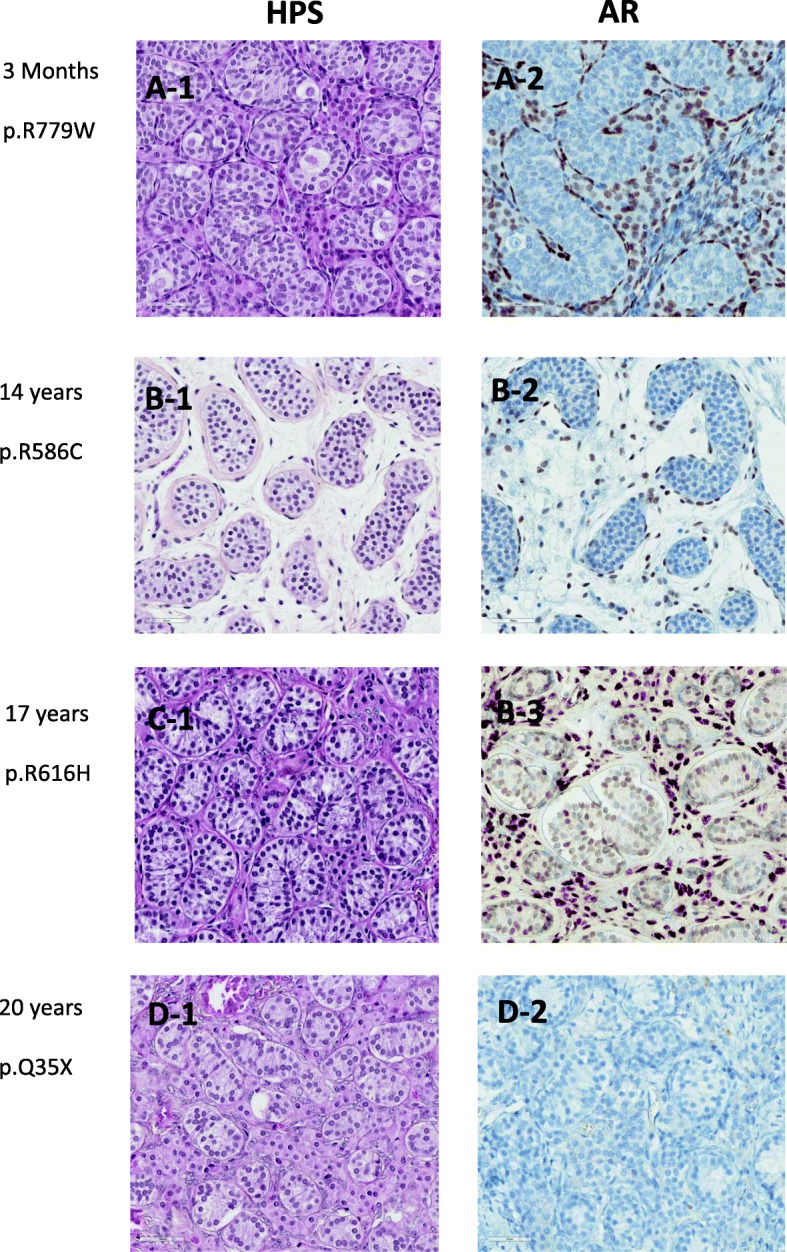
Histological analysis of testicular tissue from 4 cases of Androgen Insufficiency Syndrome (Magnification 400): age and mutations are respectively: 3 months, p.R779W (A-1; A-2), 14 years, p.R586C (B-1; B-2), 18 years, p.R616H (C-1; C-2), 20 years, p.Q35X (D-1; D-2). Hematoxylin, Phloxine, Safran (HPS): A-1; B-1; C-1; D-1 and Androgen Receptor (AR) Immunostaining: A-2; B-2; C-2; D-2

In LCT (Fig. 3 D), immunoreactivity of both AR (Fig. 3-D2) and Cx43 (Fig. 3-D3) was clearly elevated for age and AMH immunoreactivity was low for age (Fig. 3-D4). Conversely, in CAIS, immunoreactivity of both AR (Fig. 3-C2) and Cx43 (Fig. 3-C3) was undetectable in Sertoli cells and AMH ir was elevated even at pubertal age. AR ir profile in Sertoli cells was the same for the 4 cases (Fig. 5), with absence of immunoreactivity at 3 months, 14, 18 and 20 years. AR ir positive expression was observed in peritubular cells and Leydig cells for the 3 cases with point mutations (Fig. 5-A2, 5B-2, 5C-2), the inactive AR protein being recognized by the antibody. In the case with the p.Q35X mutation, AR ir was negative in all cell types, as anticipated by the early stop codon.

### Kinetics during puberty

The age of transition from prepubertal to pubertal was estimated by the point of inflexion of the sigmoid curve modeling the evolution of Sertoli cell AR ir, AMH ir and Cx43 ir, onset of a lumen in seminiferous tubules, presence of maturing germ cells, seminiferous cord/tubule diameter and S/T score (Table 3). Increased AR ir and decreased AMH ir in Sertoli cells were observed earlier than the markers of the onset of spermatogenesis. Variability between boys was given by the age of the boy with the earliest pubertal feature or value and the age of the boy with the latest prepubertal feature/value (Table 3). Large variability of age at onset of AR ir in Sertoli cells was observed: the youngest boy with clearly positive AR ir in Sertoli cells was 4.6 y.o. and the oldest boy still with negative AR immunoreactivity in Sertoli cells was 10.8 y.o. During this transition, 3/14 boys had a prepubertal feature (i.e., AR ir -; AMH ir +) and 11/14 boys had positive AR ir but with AMH ir still positive.

**Table 3:** Modeling the evolution of Sertoli cell AR ir (Immunoreactivity), AMH ir and Cx43 ir, onset of a lumen in seminiferous tubules, presence of maturing germ cells, seminiferous cord/tubule diameter and S/T score. This model could determmine ARir, AMH ir and CX ir kinetics during puberty. The age of transition from prepubertal to pubertal was estimated by the point of inflexion of the sigmoid curve modeling the evolution of Sertoli cell AR ir, AMH ir and Cx43 ir

	Age of the boy with the earliest pubertal value/feature	Age at the inflexion point of the sygmoid curve	Age of the boy with the latest prepubertal value/feature
	(Years)	(Years)	(Years)
AR ir	4.6	9.88	10.8
AMH ir	9.2	9.93	10.2
Cx43 ir	11.2	11.25	12.1
Lumen	10.8	11.42	13.8
Maturing Germ Cells	11.2	12	12.5
Cord/Tub. Diameter	11.4	12.75	13.8
S/T Score	12.1	13.5	13.8

- Cord/Tub diameter: - mean tubular diameter (cord/tubular diameter)

- S/T score: number of spermatogonia per seminiferous cord/tubule transverse section

- maturing germ cell: tubules with advanced germ cell

## Discussion

During fetal life, the gonads are structured at an early stage of embryo development (gestational week 5–8). These gonads acquire the earliest ability to produce testosterone by Leydig cells [14] and AMH by Sertoli cells [15]; both hormones are involved in male sexual differentiation. At birth, the different testicular cell types are in place but have not acquired their definitive functions [16]. During minipuberty, gonadotropic stimulation results in testosterone secretion. Despite the presence of testosterone and FSH, spermatogenesis does not progress [17]. It is thought that, because Sertoli cells are devoid of androgen receptors [18], they multiply and secrete AMH and inhibin B. Thereafter during childhood, suppression of gonadotropic secretion inhibits testosterone secretion. Inhibin B decreases during childhood [19] but Sertoli cells maintain high AMH secretion. Finally at puberty, increasing gonadotropin levels stimulate testosterone and inhibin B secretion; at this time, AMH secretion decreases [20], and the blood/testis barrier and spermatogenesis are established [21].

Initiation of spermatogenesis can be tracked by various parameters, including tubular morphological aspects and the presence of post-meiotic germ cells. In the present study, we analyzed numerous prepubertal and pubertal testicular samples with a large number of seminiferous cords/tubules for each sample. They were obtained in conditions allowing extrapolation to the physiology of testicular development from birth to puberty. Tubular diameter, S/T score, the post-meiotic germ cell and the presence of a lumen within the seminiferous tubule indicated that spermatogenesis began between 10.8 and 13.8 years. Younger subjects had only spermatogonia. Although some authors described the presence of occasional spermatocytes intended for apoptosis in some seminiferous cords during childhood [8], they were not clearly observed before the pubertal period in our control samples.

Androgens have been shown to be of major importance in the onset of spermatogenesis. This was demonstrated in an AR KO mouse model [22]. Among the effects of androgens on Sertoli cells, a decrease in AMH secretion was clearly established [17, 22, 23]. Expression of Cx43 in Sertoli cells appeared with the onset of spermatogenesis [24] and, moreover, Sertoli cell specific KO of Cx43 precluded initiation of the spermatogenic process [25]. Thus, to investigate the ability of prepubertal testicular tissues to initiate spermatogenesis in response to androgens, we studied AR, AMH and Cx43 ir according to the age group of the boys.

In all the CAIS samples (i.e., in absence of androgen effect), Sertoli cell AR ir was absent, Sertoli cell AMH ir was high and Sertoli cell Cx43 ir and spermatogenesis were absent, as previously shown [26]. But, as Chemes et al. [5] reported in a previous study on ontogeny of AR expression, we observed AR ir positive expression in peritubular cells and Leydig cells for the 3 cases with point mutation (Fig. 5A-2, 5B-2, 5C-2) allowing the inactive AR protein to be recognized by the antibody. In the case with the p.Q35X mutation, the early stop codon precluded any AR ir in any cell type. In contrast, in LCT samples, peritumoral Sertoli cells expressed AR and Cx43 ir and spermatogenesis was observed, while AMH ir was downregulated. Induction of spermatogenesis in prepubertal tissues by local production of androgens was previously shown in LCT [27] as well as in cases of testotoxicosis [28] despite the absence of FSH. Upregulation of androgens on their own receptors in Sertoli cells was demonstrated in rat [29]*.*

In the present study, the kinetics of AR, AMH and Cx43 ir and histological markers of the onset of spermatogenesis according to age demonstrated that the increase in AR ir and decrease in AMH ir in Sertoli cells were observed earlier than Cx43 ir and the histological markers of the onset of spermatogenesis. A large variability in age at onset of AR ir in Sertoli cells was observed. The youngest boy with clearly positive AR ir in Sertoli cells was 4.6 y.o. and the oldest boy with still negative AR immunoreactivity in Sertoli cells was 10.8 y.o.; at the same time, AMH ir decreased. We cannot rule out that some of the negative immune localization results were due to suboptimal tissue fixation, but we consider this unlikely to affect our main findings because of the number of different samples that we employed across the age range. The increase in nocturnal gonadotropin secretion [30] and intratesticular testosterone concentration [31] was observed at about 8 years of age, before the pubertal rise of morning blood testosterone concentration.

Our results were consistent with this timing: the significant increase in AR and decrease in AMH in Sertoli cells occurred at this time. However, Cx43 expression and onset of spermatogenesis did not occur until 11–13 years of age.

Thus, it could be anticipated that the AR content in Sertoli cells in testicular tissue obtained ahead of oncological treatment will vary according to age and to individual variations in Sertoli cell maturation. The precise time remains to be determined. AR ir was evaluated before and during culture of five human prepubertal testicular tissues [32], and increased in culture for 2 patients with low initial AR ir. Monitoring Sertoli cell maturation in culture seems to be essential for the development of culture for prepubertal testicular tissue in the hope of obtaining mature sperm cells.

Since AR ir and AMH ir have similar kinetics, Sertoli cell maturation could probably be monitored by measuring AMH in the culture medium, instead of histological evaluation of AR ir in the tissue.

Kinetically, nocturnal gonadotropin secretion [30] and intratesticular testosterone concentration [31] were observed at about 8 years of age, before the pubertal rise of morning blood testosterone concentration. The significant increase in AR and decrease in AMH in Sertoli cells occurred at this time (Fig. 2). This sequence was abolished in CAIS and was observed early in case of Leydig cell tumor in prepubertal children. Furthermore, gonadotropin or testosterone treatment or impregnation must be enough long to induce morphological change. In an experiment with human testis xenograft, 12 weeks’ treatment induced only partial modification and maturation of the somatic environment in the prepubertal testis [33], and a shorter time was observed in the only report to date of complete in vitro spermatogenesis in human adult seminiferous tubules [2].

Although this study was retrospective, limited to histological and immunocytochemical methods, used post-mortem or peritumoral tissue samples, and the analysis of cord/tubule diameter was random in the field of the microscope, we demonstrated that there was a link between androgenic stimulation, upregulation of AR in Sertoli cells, decrease in AMH in Sertoli cells and initiation of spermatogenesis. Further studies are needed to determine the precise kinetics and molecular mechanisms and Leydig cell identification.

## Conclusion

These observations highlight the fact that androgen action is required for early pubertal maturation of Sertoli cells and the beginning of the spermatogenic process in humans in vivo, similarly to what is observed in animal models. In the perspective of initiation of spermatogenesis, stimulation by testosterone will be required. The interval between androgenic stimulation, upregulation of AR and decrease in AMH in Sertoli cells and the initiation of spermatogenesis remains to be determined.

## Data Availability

The data sets generated and/or analyzed during the current study are available from the corresponding author upon reasonable request.

## Abbreviations

AMH: Anti-Mullerian Hormone
AR: Androgen receptor
CAIS: Complete androgen insensitivity syndrome
Cx43: Connexin 43
FSH: Follicle stimulating hormone
HIER: Heat-induced epitope retrieval
ir: Immunoreactivity
KO: Knock-out
LCT: Leydig cell tumor
S/T: Spermatogonia per seminiferous cord/tubule transverse section
TFI: Tubular fertility index
